# Protection from prior natural infection vs. vaccination against SARS-CoV-2—a statistical note to avoid biased interpretation

**DOI:** 10.3389/fmed.2024.1376275

**Published:** 2024-06-12

**Authors:** Susanne Weber, Pontus Hedberg, Pontus Naucler, Martin Wolkewitz

**Affiliations:** ^1^Institute of Medical Biometry and Statistics, Division Methods in Clinical Epidemiology, Faculty of Medicine and Medical Center - University of Freiburg, Freiburg, Germany; ^2^Freiburg Center for Data Analysis and Modeling, University of Freiburg, Freiburg, Germany; ^3^Department of Medicine, Huddinge, Karolinska Institutet, Stockholm, Sweden; ^4^Department of Medicine, Solna, Karolinska Institutet and Department of Infectious Diseases, Karolinska University Hospital, Stockholm, Sweden

**Keywords:** protection rate, vaccination, conditional survival, competing risks, survival of the fittest

## Abstract

**Introduction:**

The fight against SARS-CoV-2 has been a major task worldwide since it was first identified in December 2019. An imperative preventive measure is the availability of efficacious vaccines while there is also a significant interest in the protective effect of a previous SARS-CoV-2 infection on a subsequent infection (natural protection rate).

**Methods:**

In order to compare protection rates after infection and vaccination, researchers consider different effect measures such as 1 minus hazard ratio, 1 minus odds ratio, or 1 minus risk ratio. These measures differ in a setting with competing risks. Nevertheless, as there is no unique definition, these metrics are frequently used in studies examining protection rate. Comparison of protection rates via vaccination and natural infection poses several challenges. For instance many publications consider the epidemiological definition, that a reinfection after a SARS-CoV-2 infection is only possible after 90 days, whereas there is no such constraint after vaccination. Furthermore, death is more prominent as a competing event during the first 90 days after infection compared to vaccination. In this work we discuss the statistical issues that arise when investigating protection rates comparing vaccination with infection. We explore different aspects of effect measures and provide insights drawn from different analyses, distinguishing between the first and the second 90 days post-infection or vaccination.

**Results:**

In this study, we have access to real-world data of almost two million people from Stockholm County, Sweden. For the main analysis, data of over 52.000 people is considered. The infected group is younger, includes more men, and is less morbid compared to the vaccinated group. After the first 90 days, these differences increased. Analysis of the second 90 days shows differences between analysis approaches and between age groups. There are age-related differences in mortality. Considering the outcome SARS-CoV-2 infection, the effect of vaccination versus infection varies by age, showing a disadvantage for the vaccinated in the younger population, while no significant difference was found in the elderly.

**Discussion:**

To compare the effects of immunization through infection or vaccination, we emphasize consideration of several investigations. It is crucial to examine two observation periods: The first and second 90-day intervals following infection or vaccination. Additionally, methods to address imbalances are essential and need to be used. This approach supports fair comparisons, allows for more comprehensive conclusions and helps prevent biased interpretations.

## Introduction

The development of vaccines is a critical and ongoing task in the fight against coronavirus disease 2019 (COVID-19). Numerous vaccines are currently under development and some are tailored to the currently circulating Omicron sublineages. Additionally, scientists are examining the extent of protection provided by a previous severe acute respiratory syndrome coronavirus 2 (SARS-CoV-2) infection against the risk of a subsequent infection (natural protection rate). One major interest lies in the protection rates after infection compared with those after COVID-19 vaccination, e.g., (1, 2).

According to Gail et al. (3) there are different ways to measure protection rate. Vaccine effectiveness is defined as the percentage reduction in the attack rate that can be attributed to the vaccine. The attack rate is determined as the proportion of individuals infected within the designated risk group during a specified time period. This corresponds to the risk of acquiring an infection and thus the effect measure considered corresponds to 1-relative risk (RR), with RR being the relative risk of the vaccinated compared to the unvaccinated group. Additionally, Gail et al. (3) states that, when evaluating vaccine effectiveness using data from a case control study, 1-odds ratio (OR) is an appropriate effect measure, with OR being the odds ratio between the vaccinated and unvaccinated groups. In addition to these two effect measures, one minus the hazard ratio (HR) can also be considered. There are two considerations we want to point out when comparing RR, OR and HR. Considering the comparison of RR and OR a common rule is that if the event is rare (<10%) the estimates are similar, see (4). In a survival setting 1-RR and 1-HR do not differ if the hazard is small, see (3). However, in this work we focus on a competing risk setting, where the interest is in the comparison of a measure on the risk scale (RR or OR) and a measure on the rate scale (HR). In competing risk settings, the effect measures are only comparable if in addition there is no effect on the competing hazard.

When evaluating protection rates after vaccination or infection, each of these effect measures is considered. However, they address different scales. While the HR is a measure on the rate scale, the RR and the OR are measures on the risk scale. Consequently, HRs give information about direct effects on the cause-specific hazard for the event of interest and about indirect effects via influence on possible competing event hazards. For instance, death is a competing risk for the event of interest, which is infection after vaccination or infection. In contrast, ORs and RRs are summaries of direct and indirect effects, allowing for conclusions about the probability of the occurrence of the event of interest.

Although these measures differ when facing competing risks, they are all used for investigation, as there is no unique definition of the protection rate. For instance, Letizia et al. (5) used data of an observational study for investigation of the natural protection rate. Analysis was done via Poisson regression and 1-HR is reported. Dagan et al. (6) investigated vaccine effectiveness using Kaplan–Meier estimators and used the corresponding risk estimates in order to obtain the vaccine effectiveness via 1-RR. Powell et al. (7) considered 1- odds ratio (OR) in order to compare protection rate after infection and vaccination for different variants.

It should be noted, that when considering former SARS-CoV-2 infection as an exposure and its effects, the competing risk of death is more prominent than after COVID-19 vaccination. The infection affects the mortality hazard, which has an impact on the time at risk for developing a further infection and is hence indirectly affecting the infection risk. Furthermore, in publications a reinfection after a SARS-CoV-2 infection is only possible after 90 days per epidemiological definition (1, 2). Hence, analysis of the protection rate of an infection starts after 90 days and only individuals surviving the first 90 days are at risk of a reinfection. In contrast, there is no such constraint for the analysis of protection rate after vaccination. Consequently, when comparing protection rate after infection or vaccination, analysis should start after 90 days in order to avoid immortal time bias. However, this is not a fair comparison, as it is prone to selection bias. The mortality hazard increases in the initial period after an infection and subsequently decreases until 90 days post-infection. Thus, as elderly and more morbid patients are at a higher risk of dying due to infection during the first 90 days, the population is overall healthier during the second 90 days. For the vaccinated group there is no such selection during the first 90 days, as the vaccination does not affect the mortality hazard.

Thus, comparison of the protection rate of vaccination versus natural infection poses several challenges due to significant differences in reinfection and death rates among groups within the first 90 days.

To examine the statistical challenges that arise from assessing protection rates, we have access to population-based observational data from several databases from Stockholm County, Sweden. Information on almost two million people is available from 2020 to 2022. Information about SARS-CoV-2 infections and vaccinations is provided, alongside other patient-related characteristics, all derived from population-based data sources with high coverage. Thus, when comparing the natural protection with protection after a vaccination, we can examine imbalances between groups at baseline and during follow-up and discuss solutions to address them. Furthermore, we estimate HRs and ORs for comparison, in order to obtain one measure on the rate scale and one measure on the risk scale.

For a comparison between immunization via infection or vaccination, we consider several investigations. We highlight different aspects of effect measures and insights drawn from different analyses. The aim is to promote an awareness of the differences between the causes of protection in order to create a fair comparison.

## Materials and methods

### Study population

Among the entire population of Stockholm County, Sweden, we identified all individuals born 2001 or earlier, thus being 18 years or older during the entire COVID-19 pandemic period. We included all individuals alive and residing in Stockholm County on the 15th of March 2020. Individuals with a PCR test positive for SARS-CoV-2 before the 16th March 2020 were excluded.

### Data sources

Data were linked from three population-based data sources using personal identification numbers, unique for each Swedish resident, from the Stockholm regional healthcare data warehouse (VAL), SmiNet, and the National Vaccination Register (NVR). VAL contains data from administrative healthcare databases within the Stockholm Region, including demographics, migration, drug prescriptions, and data on all inpatient stays and outpatient visits reimbursed by Region Stockholm (8). This includes near complete coverage of specialist care and 94% of primary care (8). SmiNet contains all PCR SARS-CoV-2 positive test results reported in accordance with the Communicable Diseases Act (9). The data from NVR included all COVID-19 vaccinations administered in Sweden to the Stockholm County population.^1^

### Analysis

The aim of this work is the comparison of protection of a SARS-CoV-2 infection after a first vaccination without being infected before, or a first infection without being vaccinated before. For simplicity, we will call the first vaccination or infection “*time of first immunization*.”

We perform several analyses. First, we determine the inclusion window in order to define the study population for the main analysis of this work. The main analysis addresses the comparison of protection rate after vaccination with the natural protection rate. Observation for this main analysis starts at time of first immunization. Thus, the focus of the first analysis is to investigate the time to first immunization and in the main analysis, we focus on challenges concerning group imbalances and different effect measures. We distinguish between the first and the second 90 days after first immunization and investigate both observation periods.

#### Determination of study cohort for main analysis (inclusion window)

As we want to compare protection rates after first vaccination and first SARS-CoV-2 infection, we have to define an inclusion window in order to define a study cohort with reasonable groups for comparison. Thus, we first have a look at the competing risk model considering time to first immunization (first vaccination or first SARS-CoV-2 infection separately). Death is a competing risk, see Supplementary Figure S1. The aim is to define an inclusion window, so that the vaccinated group and the infected group are both big enough and facing the same pandemic situation.

Follow-up for this analysis starts on 2020-03-15 when a more extensive transmission of the virus started. Note, that vaccination first was possible on December 27th 2020 in Sweden.

#### Main analysis

In order to investigate protection rate after immunization via SARS-CoV-2 infection or vaccination, we distinguish between two observation periods. The first observation period represents the first 90 days after the time of first immunization. The second observation period represents the second 90 days, starting at day 91. In general a reinfection after SARS-CoV-2 infection is per definition only possible after 90 days, thus investigation of the protection rate starts after the first 90 days. The first 90 days represent the selection process, selecting individuals who are surviving the first 90 days and are thus available for the main analysis. Hence, the first 90 days after immunization, as well as the second 90 days should be considered and investigated.

Time zero is the time of the first immunization. In the following, the groups for comparison of the protection rate after SARS-CoV-2 or vaccination are called the infected and the vaccinated group, respectively. The infected group will be considered as the reference group. Possible confounders measured at time zero are age (continuous), sex (binary), and comorbidity count (categorical). Comorbidity count has categories 0,1,2,3, ≥ 4 and considers the following comorbidities: cancer, cardiac disease, cerebrovascular disease, chronic kidney failure, chronic liver disease, chronic lung disease, dementia, diabetes, dialysis, down syndrome, hypertension, mental health disorder, mental retardation, neurological disease, obesity, other immunocompromising conditions and treatments, pregnancy, transplantation (solid organ or stem cell), living in nursing home, and receiving home help services.

During the first 90 days, people in the vaccinated group can get an infection or they can die without an infection. In contrast to that, people in the infected group can die during the first 90 days and they can be vaccinated for the first time, but they cannot get infected [per definition; see (1, 7)]. Thus, groups are not comparable concerning reinfection during first 90 days.

Selection for the second analysis occurs at the end of the first observation period. Available for the analysis of the second 90 days are those people still alive and without an infection at the end of the first observation period, and without first vaccination after having the first SARS-CoV-2 infection. The outcome of interest during the second observation period is the occurrence of first SARS-CoV-2 after immunization during the second 90 days.

The second analysis is a conditional analysis. The first analysis provides information about the selection process for this conditional analysis.

#### Analysis of the first 90 days: selection for conditional survival

The chosen model is a competing risks model with three possible events during a follow-up of 90 days: first SARS-CoV-2 after immunization via first vaccination, Death, and first Vaccination after Immunization via first SARS-CoV-2 (see Supplementary Figure S2).

Note that we do not handle second vaccination as competing risk in the vaccination group. For simplicity, we decided that a further vaccination is no reason for exclusion of analysis of the second 90 days. Due to the methodological character of this work, we think that this is a reasonable choice.

The estimated transition probabilities (via the etm package in R) are illustrated via stacked probabilities plots. Death (death without first Covid19 nor first vaccination, and death overall) during the first 90 days is investigated via Cox regression and logistic regression. Continuous baseline characteristics are given by mean, standard deviation (SD), median, first quartile (Q1), and third quartile (Q3). Categorical baseline characteristics are given by percentages. A comparison has been done of the baseline characteristics for the baseline population and the population selected for the conditional survival analysis.

#### Analysis of the second 90 days: conditional survival

For the conditional survival analysis, follow-up starts 90 days after first immunization, see the competing risks model as depicted in Supplementary Figure S3. Follow-up is 90 days and the possible events are first SARS-CoV-2 infection after immunization or death. Excluded from the analysis are individuals who had a SARS-CoV-2 infection, who are first vaccinated, or who died within the first 90 days.

Conditional survival is actually what is being done in the literature when considering natural protection rate. In these examples, the first 90 days after infection are not considered (at the most briefly in the discussion, e.g., (1, 10)).

We estimate two effect measures considered in the literature when investigating the protection rate: HR and OR. Note that usually 1 minus the respective effect measure is given. The infected group is considered as the reference group. HRs are estimated via the survival package in R, ORs are estimated via the glm function using logit as a link function.

Focus of this analysis are differences between the effect measures and imbalances between the groups.

Both a crude analysis, i.e., without any adjustment, and several approaches, addressing the imbalance between groups were done: regressions with adjustment for baseline covariates (age (continuous), sex (binary), comorbidity count (categorical)), analysis of matched cohorts (matching of selected population via the same baseline covariates using the matchit package in R), and one weighted analysis.

Matched logistic regression is done using a mixed model with the matching group as a random effect (via lme4 package in R). The matching approach performs generalized full matching in the selected population. This is a faster alternative to the full matching approach and thus applicable to a large dataset. This matching approach estimates the average treatment effect (ATE) in a population compared to the selected population available after 90 days.

The weighting approach considers the selected population and uses inverse probability weights obtained via the weightit package considering the ATE option and each of the covariates mentioned above.

An overview of the different analysis approaches is listed in Table 1.

**Table 1:** Different analysis approaches for investigating protection rate and how imbalances between groups are addressed.

Approach	How?	What is addressed?
Crude		crude group comparison ignoring imbalances
Adjusted		conditional effect in selected population addressing imbalances after selection
Matched	Generalized Full Matching (ATE in selected population)	addressing imbalances after selection (Generalized full matching is often faster than even nearest neighbor matching, especially for large datasets)
Weighted	ATE in selected population	addressing imbalances after selection

ATE, average treatment effect.

Analysis is done in R (Version 4.1.0).

## Results

### Study population

On March 15, 2020, 1,860,797 subjects were available in the dataset according to the inclusion criteria. A small proportion of those (*N* = 11,203, 0.6%) were excluded as there were some inconsistencies with the data during the follow up, for example earlier death date than first SARS-CoV-2 infection, second SARS-CoV-2 infection, or first vaccination. Thus, for the first analysis, the determination of the inclusion window for the time-to-event analysis resulted in 1,849,594 available subjects. In the case where the death date equaled the infection or vaccination date, 0.001 was added to the time of death.

**Figure 1:**
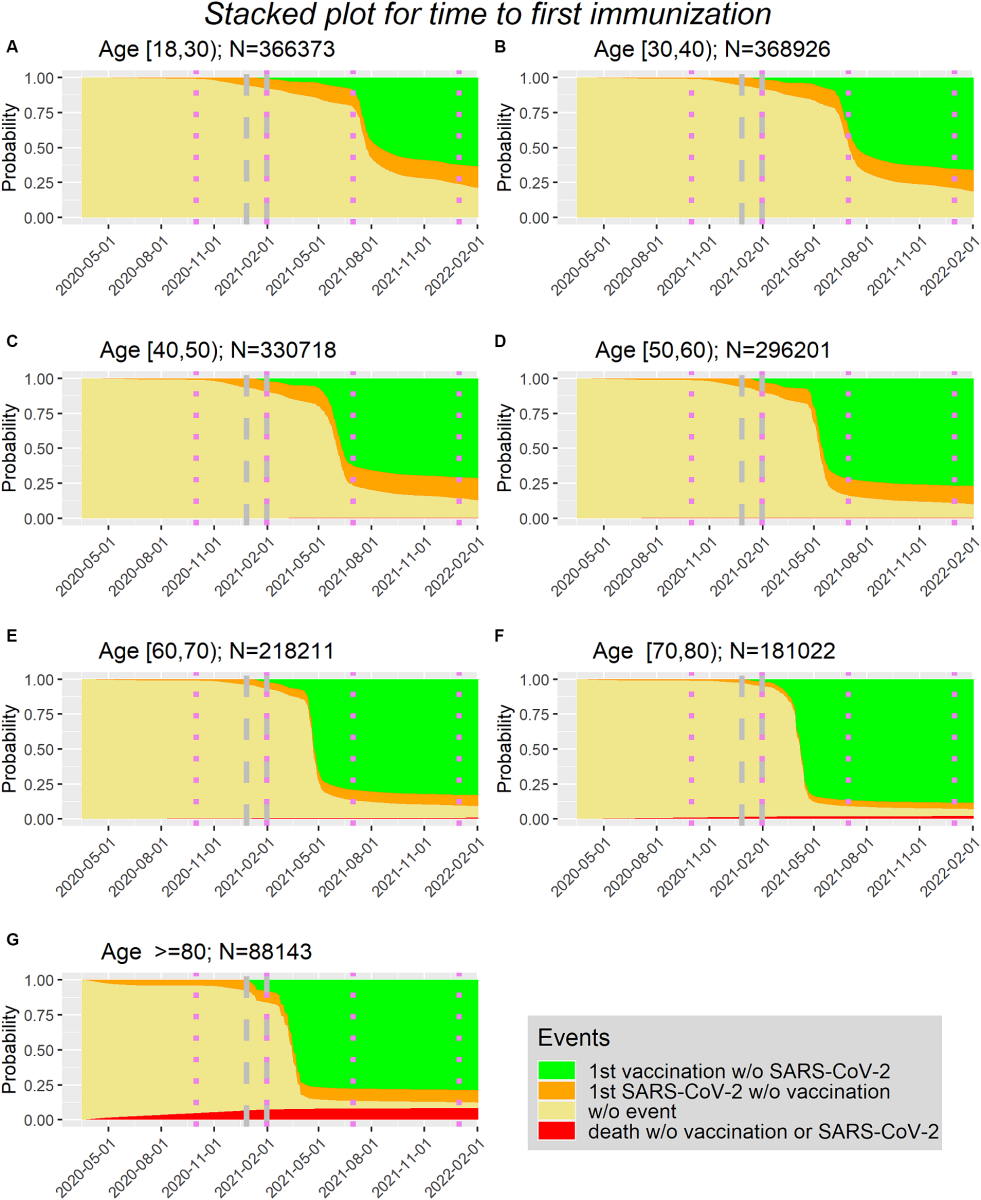
**(A–G)** Time to first immunization via first vaccination or first SARS-lines show borders of surges in Sweden in age groups (10 year steps, with **(A)** being the youngest group (Age between 18 and 30) and **(G)** being the oldest group (Age>=80)).

Figure 1 shows the stacked plots in age groups (10 year steps) with the cumulative transition probabilities corresponding to the competing risk model addressing the time to first immunization.

It can be seen that vaccination is first possible at the end of 2020 (27 December 2020). Furthermore, in the orange area, which represents the proportion of people being first infected without being vaccinated before, there is only a minor increase after the beginning of 2021.

According to the National Board of Health and Welfare (11) the first four surges in Sweden are as follows: 1. from March to September 2020, 2. from October 2020 to January 2021, 3. from February to June 2021, 4. from July to December 2021. These boundaries can be seen via the violet dotted lines in Figure 1.

With this information and the development of the curves in Figure 1, the inclusion window for the study population is defined as first immunization between 2020-12-27 and 2021-01-31 (dates included). Using these dates implies that for the analysis of the first 90 days the follow-up of 90 days might fall into two surges (period 2 and 3). For the analysis of the second 90 days the follow-up of 90 days lies completely in period 3.

### Analysis of first 90 days

According to the inclusion window, the time of first immunization lies between 2020-12-27 and 2021-01-31 for 56,201 subjects.

The baseline characteristics (as available in December 2020 for each subject) are given in Table 2.

**Table 2:** Baseline characteristics for the population at baseline and for the selected population available after the first 90 days.

	Population at baseline		Population after 90 days	
	Infected group (*N* = 19,335)	Vaccinated group (*N* = 36,866)	Infected group (*N* = 16,924)	Vaccinated group (*N* = 35,525)
Age				
Mean (SD)	44.4 (16.9)	56.7 (20.4)	41.7 (14.3)	56.0 (20.1)
Median [Q1,Q3]	43.0 [31.0,55.0]	55.0 [41.0,73.0]	40.0 [30.0,52.0]	54.0 [41.0,71.0]
Sex				
Female	9,990 (51.7%)	26,106 (70.8%)	8,570 (50.6%)	25,223 (71.0%)
Male	9,345 (48.3%)	10,760 (29.2%)	8,354 (49.4%)	10,302 (29.0%)
Comorbidity count				
0	14,376 (74.4%)	19,623 (53.2%)	13,443 (79.4%)	19,381 (54.6%)
1	2,891 (15.0%)	5,886 (16.0%)	2,406 (14.2%)	5,762 (16.2%)
2	1,077 (5.6%)	4,018 (10.9%)	701 (4.1%)	3,769 (10.6%)
3	530 (2.7%)	3,561 (9.7%)	251 (1.5%)	3,261 (9.2%)
> = 4	461 (2.4%)	3,778 (10.2%)	123 (0.7%)	3,352 (9.4%)

The infected group is younger, has more men and is less morbid compared to the vaccinated group.

**Figure 2:**
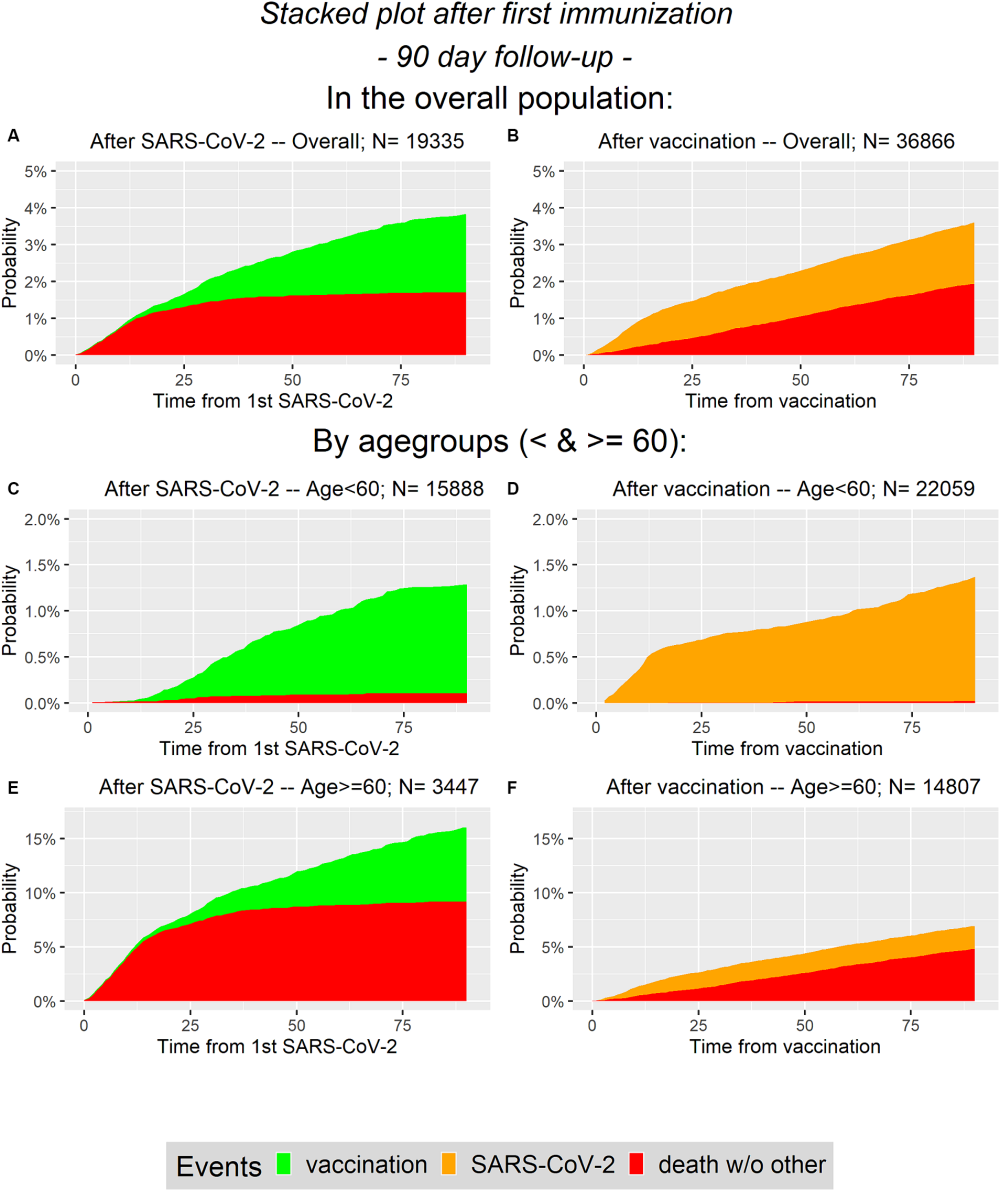
Cumulative probability plots for analysis of first 90 days. On the left panel **(A,C,E)** the infected group is presented and on the right panel **(B,D,F)** the vaccinated group is considered. In the first line **(A,B)** the overall population is presented, while the second and the third line represent the age groups (**C–F**, respectively).

In Figure 2 the cumulative incidences correspond to the competing risks model addressing the first 90 days after first immunization for the overall population and in age groups (<60 and ≥ 60).

Note that the ranges of the y-axis differ depending on the considered population (overall or in age groups). Obviously, one difference is that during the first 90 days there are no SARS-CoV-2 infections in the infected group, i.e., there are no orange areas in the left column. In contrast, there are no green areas on the right column. Recall that vaccination is not considered as competing event in the vaccinated group as we do not distinguish between different vaccination states. It can be seen, that the selected proportions for the conditional analysis in the overall population are similar between groups. The probability of having an event during the first 90 days is between 3.5 and 4% in both groups. In contrast to that, in the elderly population it is more likely to survive the first 90 days without an event in the vaccinated group, compared to the infected group. There is a difference between the two groups concerning the competing event death, especially in the elderly.

We performed regression analysis of death without other events during the first 90 days via Cox and logistic regression in the overall population and in age groups. In Table 3, the results are presented for crude regression and with adjustment for sex, age (continuous), and comorbidity count (categorical).

**Table 3:** Results of regression analysis for death without other event during the first 90 days after first immunization in the overall population and in age groups.

		Death without other event (vaccination vs. infection)	
Population		HR	OR
Overall	Unadjusted	1.118 [0.981;1.274]	1.153 [1.011;1.316]
	Adjusted	0.166 [0.144;0.192]	0.216 [0.184;0.253]
<60	Unadjusted	0.222 [0.081;0.607]	0.225 [0.074;0.574]
	Adjusted	0.153 [0.055;0.429]	0.185 [0.059;0.482]
≥60	Unadjusted	0.47 [0.411;0.537]	0.509 [0.444;0.585]
	Adjusted	0.171 [0.148;0.198]	0.31 [0.267;0.361]

The infection is considered as the reference group. HR, hazard ratio; OR, odds ratio.

It is strikes that the unadjusted HRs and ORs are smaller than one in both age groups, but greater than one in the overall population. This situation is known as the Simpson Paradox (12). The reason therefore is that the sample size of the two groups in the age groups differ. The elderly infected group is only a small proportion of the overall SARS-CoV-2 population (3,447 of 19,335, 17.8%), whereas this is not the case for the vaccinated group (14,807 of 36,866, 40.2%). But, most of the deaths occur in the elderly population (in both immunization groups).

Adjusting or at least considering age groups leads to estimated effect sizes clearly apart from one, i.e., the unadjusted ORs by age groups are 0.225 for the younger population and 0.509 in the elderly.

**Figure 3:**
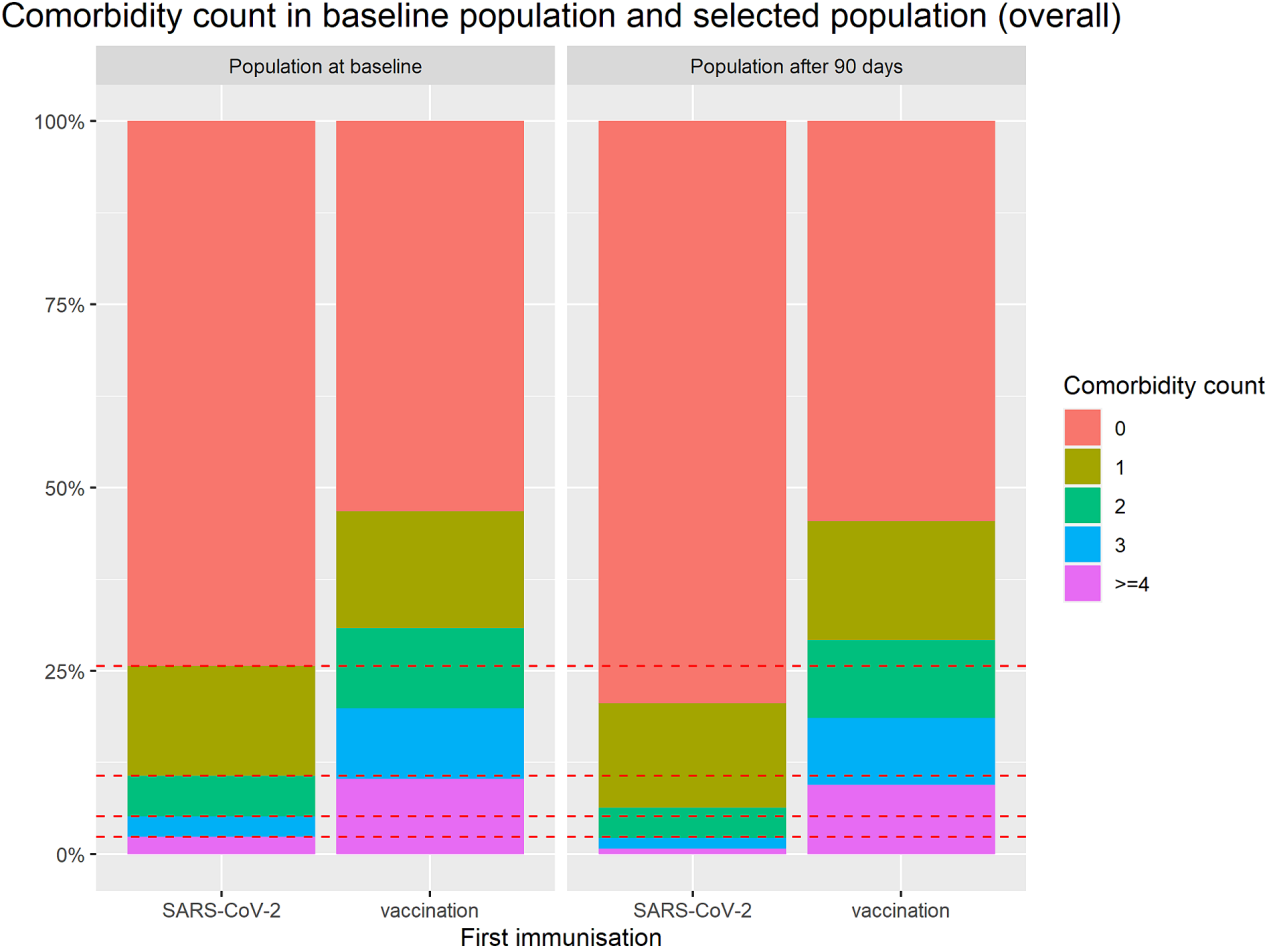
Comparison of comorbidity count between groups for baseline population and selected population available for conditional analysis after first 90 days.

In order to see how this selection process affects the study population the baseline characteristics for the selected population are also listed in Table 2. It can be seen that the differences in age and comorbidity count even increased in the available population. While the vaccinated group has only minor changes in these two variables, the infected group, notably, became less morbid from the first 90 days to the second 90 days, as more morbid patients passing away. Out of 461 subjects with a comorbidity count of ≥4 only 123 survived the first 90 days without an event. Note that in the vaccination group 3,352 of 3,778 with ≥4 comorbidities survived the first 90 days without an event. This difference is illustrated in Figure 3.

Note that the different movements in immunization groups mainly occurs in the older population, see Supplementary Figure S4 and Supplementary Table S1.

In conclusion, the population is changing within 90 days in a different way in the both groups. For the analysis of the second 90 days addressing the protection rate, the groups need to be made comparable in order to make a fair comparison.

### Analysis of second 90 days

In order to see how the matching and weighting mechanisms perform, love plots are shown in Supplementary Figure S5.

**Figure 4:**
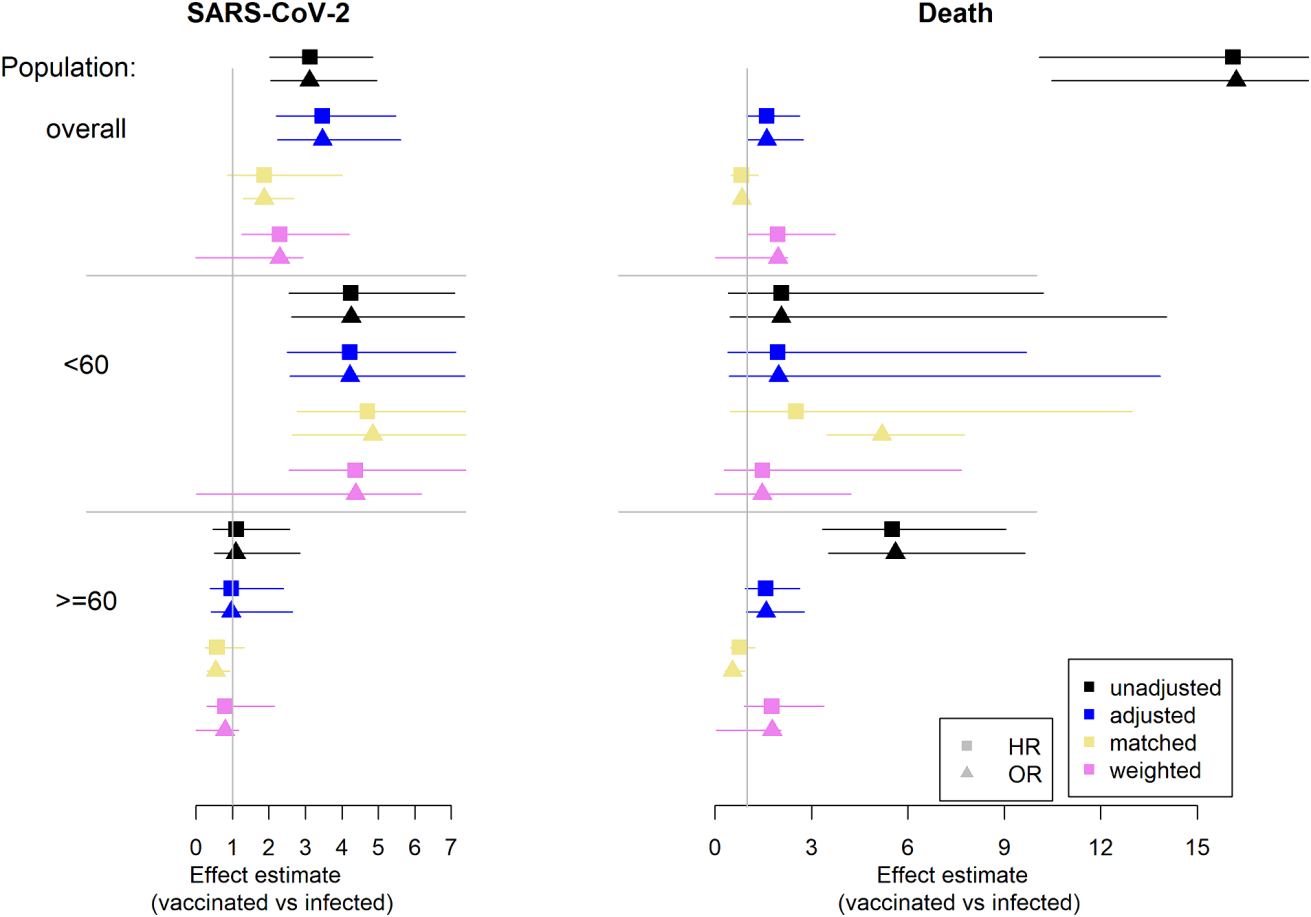
Estimation of effect estimates (HR and OR): results from regression analysis. Infection is considered as reference group. On the left column the outcome SARS-CoV-2 infection is considered and on the right column the competing event death is considered (HR, hazard ratio, OR, odds ratio).

In Figure 4, the results of the different regression analyses are presented. The exact values are listed in the Supplementary Table S2.

Note that adjustment is for sex, age (continuously), and comorbidity count (categorical).

The unadjusted analysis considering death shows HRs and ORs of with values greater than 12 in the overall population and values greater than 5 in the elderly. However, this is not a fair comparison. Recall the imbalances in the considered population. The vaccinated group is older and has more comorbidities. These are two factors with an impact on death. The estimated effect can be mainly explained by these group differences. This can be seen by the effect estimates for the different approaches addressing imbalances.

It strikes, that there is a difference in age groups. In the younger population there is no difference in the approaches and the confidence intervals are wide. Note, that there are only very few deaths in this population, similar in both groups. However, looking at the older population there are some minor differences between the approaches. Most of the deaths occur in this population. The increase in imbalances between groups after selection mainly comes from the elderly. In this population the infected group is more robust due to frailer patients dying in the initial 90 days after infection. While there is no such effect in the vaccinated group (see Supplementary Figure S4). Hence, the huge unadjusted effect in the overall population mainly comes from the older population. Addressing the imbalances via the different approaches reduce the estimated effect.

Considering the outcome SARS-CoV-2 infection, there is mainly a difference between age groups. In the overall population there is an effect of vaccination vs. infection throughout the approaches (not always statistically significant). This effect comes from the younger population. In this population the vaccinated group has a disadvantage compared to the infected group. This is in contrast to no difference being found in the elderly population.

Concerning the issue of competing risks there are no big differences between the estimates of the HR and the OR. Hence, the topic of competing risks is not a big issue (at least in this data example). Note that the event rates during the second 90 days are low in each age group and for each event.

## Discussion

In this work we investigated challenges when comparing the protection rate for SARS-CoV-2 infection after an infection or after vaccination. We divided the time after first immunization into the first and the second 90 days of follow-up. Due to the epidemiological definition, a comparison of protection rates after infection or vaccination has to start after the first 90 days. This results in a selection process for the analysis. We have seen that this selection process differs between groups. While in the vaccinated group there were not many changes concerning the distribution of the baseline covariates in the selected population compared to the baseline population, in the infected group there were substantial differences. The selection process in the infected population resembles a “survival of the fittest” scenario. The two groups (vaccinated and infected) already had imbalances at baseline and the selection process intensifies this.

The considered approaches - adjustment, matching and weighting - obviously cannot address the selection process itself in an explicit way, as only the selected population is considered. However, it is important to consider the differences in mortality during the first 90 days when reporting the effects after 90 days. This will enable decision-makers to make a careful risk assessment.

We strongly recommend not to only start the analysis after 90 days and ignore the first 90 days, but rather investigate the selection process itself.

There are numerous approaches to address the imbalances between groups. In this work we presented adjustment, general full matching, and weighting addressing ATE as an estimand. Of course there are more possibilities to address imbalances in regression analyses. For instance, there are already several ways of performing matching or weighting, see (13). When choosing a method, one should be aware of what estimand is being addressed, in order to interpret the resulting estimation. It is crucial to be aware of differences between groups and addressing them in the analysis. Especially if the group comparison considers a vaccinated group versus a non-vaccinated group, it is possible that the data is prone to the healthy vaccine bias, see (14, 15). This bias occurs if the vaccinated group is in general more healthy than the comparison group. One way to investigate the healthy vaccine bias is comparing the non-COVID mortality in both groups. However, in order to do this, the cause of death needs to be known. Note, that in our data, the infected group, which is the non-vaccinated group, is less morbid than the vaccinated group, based on age and comorbidity count at baseline.

Furthermore, the competing risk has to be taken into account. Using data from Stockholm, the impact of the competing risk did not lead to a large discrepancy between ORs and HRs. An explanation might be that there are only few infections during the second 90 days, and even fewer deaths. Hence the event rates are low for each of the competing events. However, from a patient’s point of view it is important to get information about both events. Therefore, we advise to always report both, i.e., measures on rate and on the risk scale, for the outcome and the competing event death in order to get a complete picture of the risk dynamic.

For our investigation we were able to use an extensive dataset from Stockholm County with information on over 1.8 million people. This allowed us to consider only a small inclusion window for this study and still have a sample size of over 52.000. With this inclusion window we ensured that there was no notable variation in the virus at time of immunization and in the time considered for infection after immunization (i.e., the second 90 days after immunization).

A limitation of this investigation is that we did not distinguish between different levels of vaccination. For simplicity we only considered vaccinated as being at least vaccinated once and without previous infection. For the illustrative purpose of this work, this is a justifiable simplification. Allowing for more complexity in the determination of immunization groups requires more thought concerning the time at risk considered in the analysis comparing the groups. However, since the analysis is limited to short time frames (the first and second 90-day periods after initial vaccination), the consideration of extra doses is of minor importance.

Furthermore it needs to be noted, that we did not distinguish between SARS-CoV-2 related deaths and non-related deaths. While this differentiation is not important for the purpose of this work, it is quite important for clinicians and patients and should be incorporated when investigating related research questions.

It is important to note that infections in this dataset are only identified when individuals are tested. Unfortunately, we do not have data on testing frequencies. Hence we cannot compare them between the infected and the vaccination group in order to see whether this is similar.

Even though the progress of the pandemic has led to changes in the underlying populations, the topic of this work remains relevant. Over time, the number of people with numerous infections and vaccinations has increased. Hence the analysis has become more complex. Nevertheless, the initial problem is still present. If the main analysis starts after 90 days, and thus there is a selection process during the first 90 days, it is crucial to take this first period into account as there might be differences between groups. Hence it is necessary to evaluate this problem in a simple setting in order to get a better understanding.

In conclusion, for a comparison between immunization via infection or vaccination, we strongly emphasize to consider several investigations in order to make fair comparisons and to draw comprehensive conclusions. Information on the selection process for the main analysis should be investigated and reported in the publication, namely the first 90 days. It is essential to present both in order to avoid biased interpretation.

## Data availability statement

The data analyzed in this study is subject to the following licenses/restrictions: the individual participant data underlying this article were subject to ethical approval and cannot be shared publicly. Data from the deidentified administrative health registries are not freely available due to protection of the personal integrity of the participants. Requests to access these datasets should be directed to PN, pontus.naucler@ki.se.

## Ethics statement

The studies involving humans were approved by Swedish Ethical Review Authority (Dnr 2018/1030–31, COVID-19 research amendments Dnr 2020–01385, 2020–02145, 2020–04069 and 2022–02127-02). The studies were conducted in accordance with the local legislation and institutional requirements. The ethics committee/institutional review board waived the requirement of written informed consent for participation from the participants or the participants’ legal guardians/next of kin because the need for consent was waived by the Swedish Ethical Review Authority (Dnr 2018/1030–31, COVID-19 research amendments Dnr 2020–01385, 2020–02145, 2020–04069 and 2022–02127-02) since analyses are based on retrospectively collected data from the administrative health registry.

## Author contributions

SW: Conceptualization, Formal analysis, Investigation, Methodology, Visualization, Writing – original draft, Writing – review & editing. PH: Conceptualization, Data curation, Formal analysis, Writing – original draft, Writing – review & editing. PN: Conceptualization, Supervision, Writing – original draft, Writing – review & editing. MW: Conceptualization, Project administration, Supervision, Writing – original draft, Writing – review & editing.

## Footnotes

^1^ https://www.folkhalsomyndigheten.se/the-public-health-agency-of-sweden/communicable-disease-control/vaccinations/vaccination-register-and-vaccination-coverage/variable-list-for-the-national-vaccination-register/
