# Supplementary material for: Protection from prior natural infection vs. vaccination against SARS-CoV-2—a statistical note to avoid biased interpretation
Source: Front Med (Lausanne). 2024 Jun 12;11:1376275. doi: 10.3389/fmed.2024.1376275 (PMC11199770; doi:10.3389/fmed.2024.1376275)
Supplement: Supplementary file 1 [file Data_Sheet_1.pdf]

Supplementary Material

Protection from prior natural infection versus vaccination against SARS-CoV-2 – a statistical note to avoid biased interpretation.

Susanne Weber1,2,*, Pontus Hedberg3 , Pontus Naucler4 and Martin Wolkewitz 1,2

*** Correspondence:** Susanne Weber: Susanne.weber@uniklinik-freiburg.de

1 Supplementary Figures and Tables

1.1 Supplementary Figures

Figure S1 Competing risks setting considered for determination of inclusion window for study cohort of main analysis.

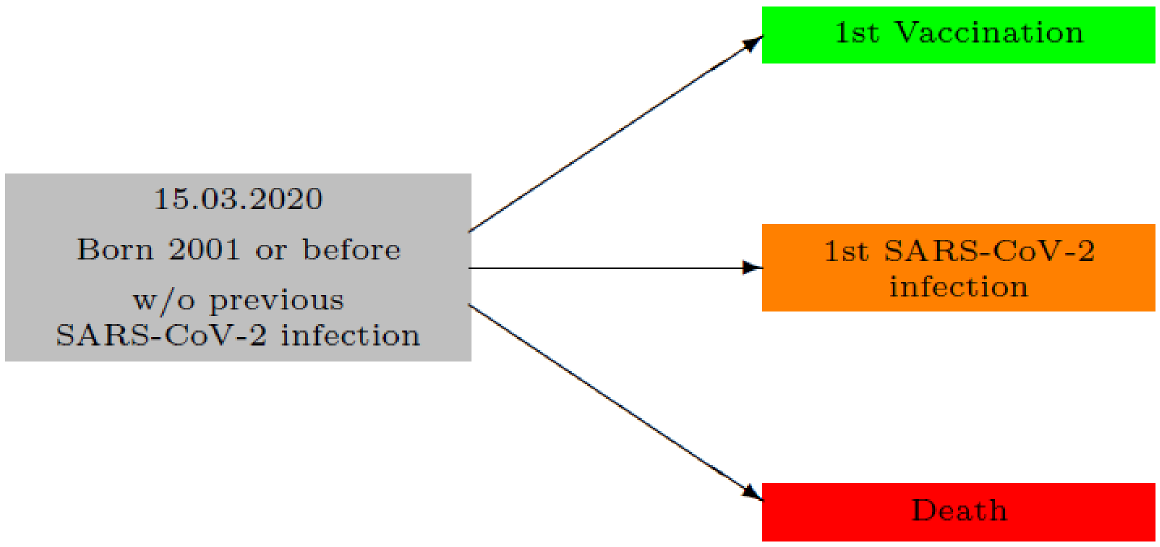

Figure S2 Competing risks setting considered for investigation of 1st 90 days after immunization. After 1st immunization via vaccination a person can get either infected or die. After 1st immunization via a SARS-CoV-2 infection a person can either die or be 1st vaccinated.

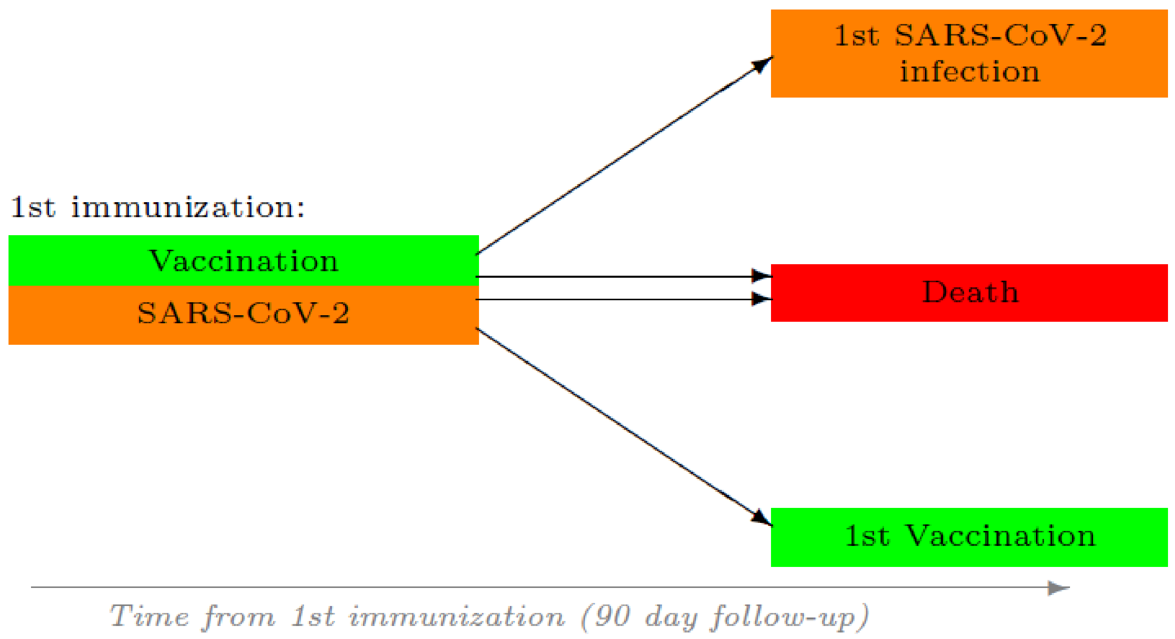

Figure S3 Competing risks setting considered for investigation of the 2nd 90 days after immunization. Possible outcomes for both immunization groups are 1st SARS-CoV-2 infection after immunization or death.

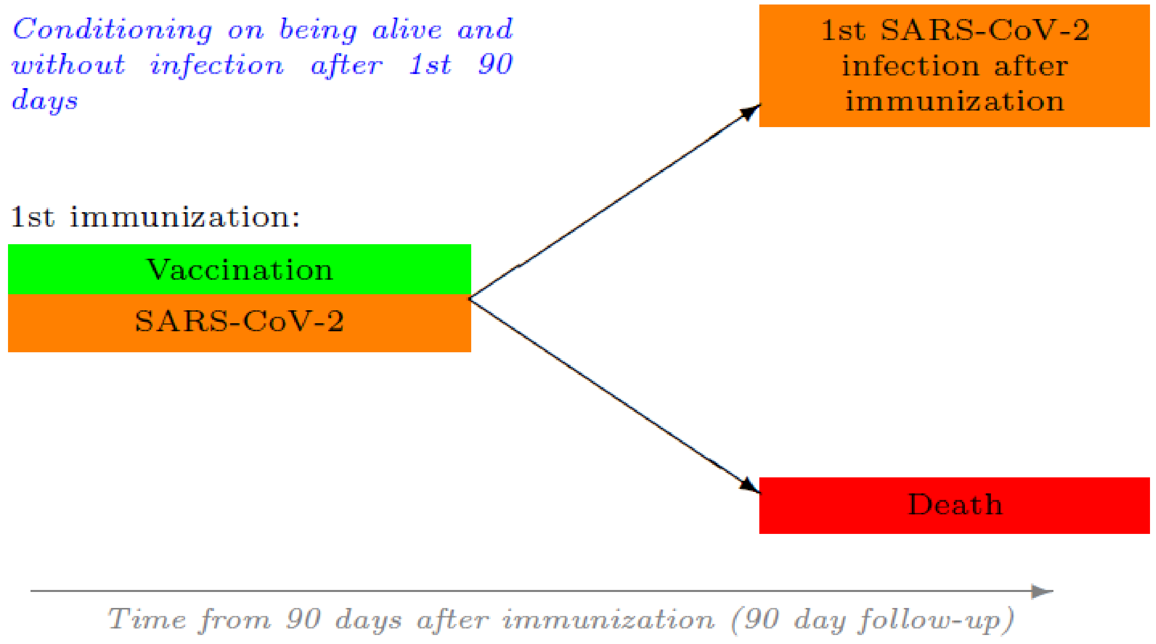

Figure S4 Illustration of differences in comorbidity count comparing the immunization groups at baseline and after selection of the available population for the 2nd 90 days.

(A) Overall population.

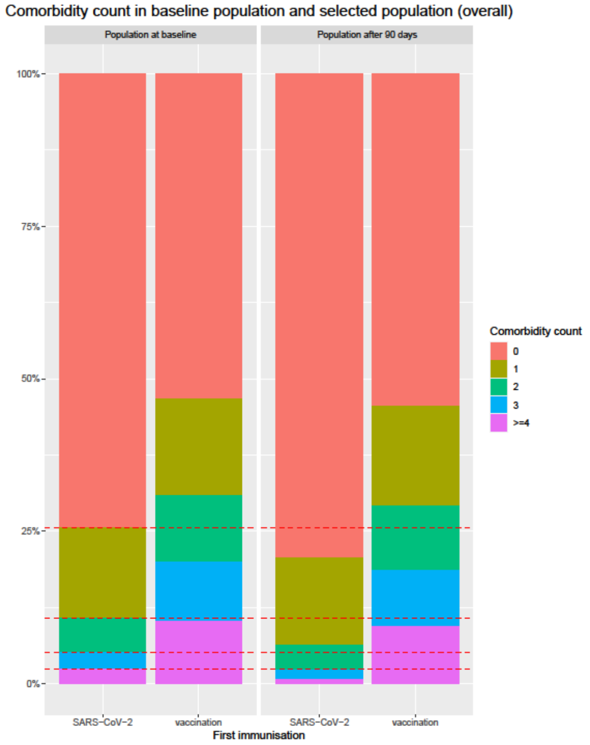

(B) Younger population with age <60.

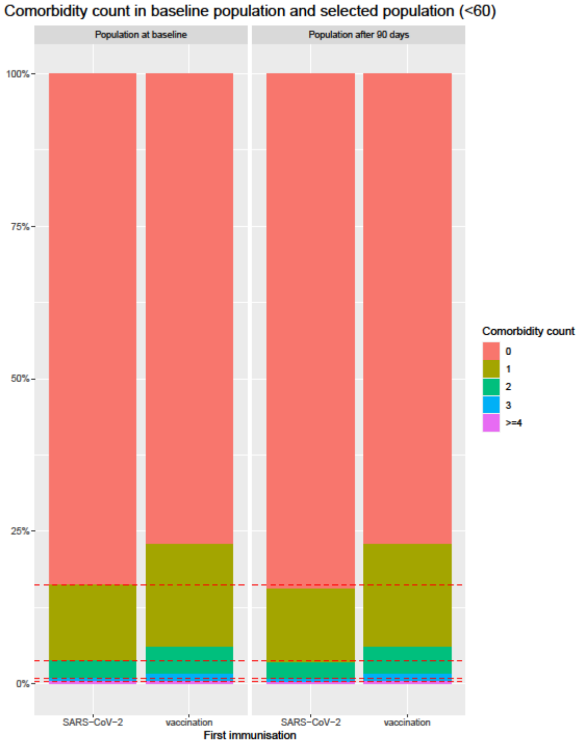

(C) Older population with age >=60.

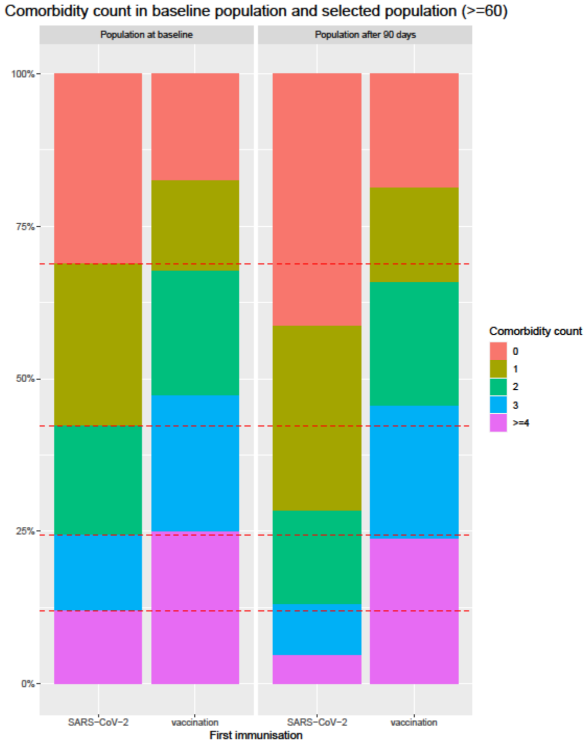

Figure S5 Love plots for different approaches.

A) Matching: Group differences before and after matching.

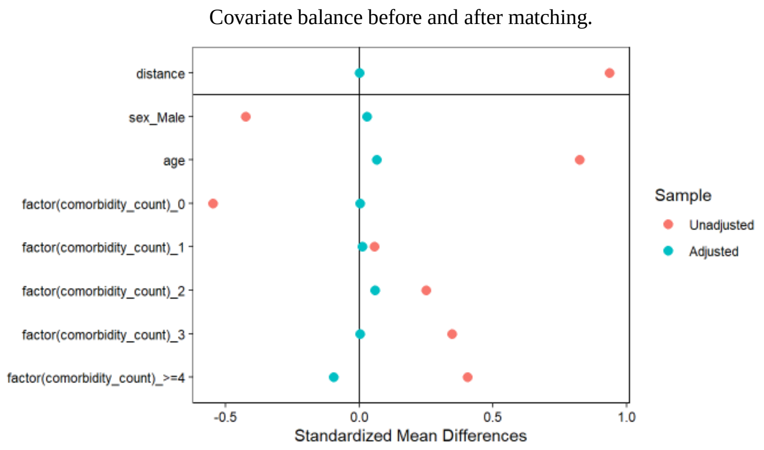

B) Weighting: With and without weights.

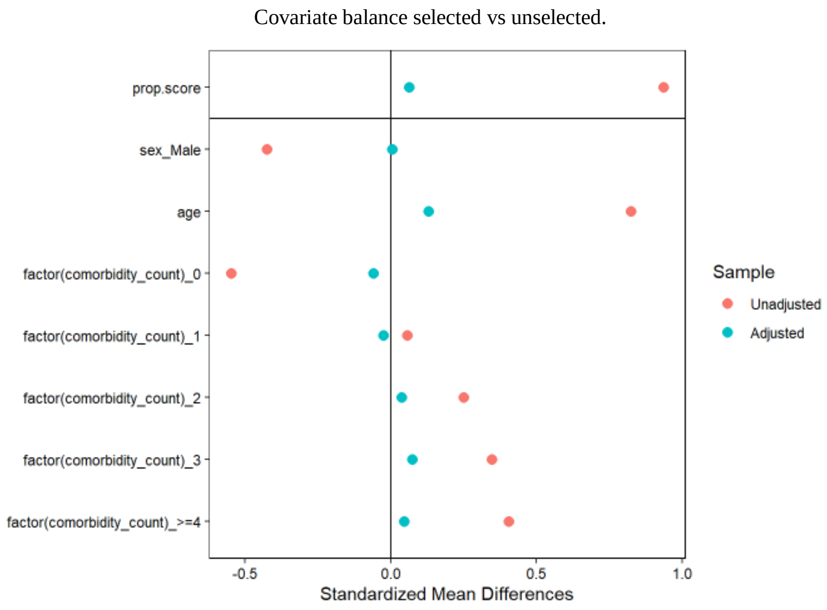

1.2 Supplementary Tables

Table ST1 Comparison of baseline characteristics in age groups for population at baseline and for the population available after the 1st 90 days.

Baseline Characteristics -- for younger population (<60) available at baseline and after 90 days

	Population at baseline		Population after 90 days	
	C19 (N=15888)	vaccination (N=22059)	C19 (N=14926)	vaccination (N=21752)
**age**				
Mean (SD)	38.5 (11.4)	42.8 (11.1)	38.3 (11.4)	42.8 (11.1)
Median [Q1,Q3]	38.0 [29.0,48.0]	44.0 [34.0,53.0]	38.0 [29.0,48.0]	44.0 [34.0,53.0]
**factor(sex)**				
Female	8232 (51.8%)	16012 (72.6%)	7593 (50.9%)	15781 (72.5%)
Male	7656 (48.2%)	6047 (27.4%)	7333 (49.1%)	5971 (27.5%)
**factor(comorbidity_count)**				
0	13304 (83.7%)	17017 (77.1%)	12617 (84.5%)	16797 (77.2%)
1	1975 (12.4%)	3702 (16.8%)	1800 (12.1%)	3640 (16.7%)
2	460 (2.9%)	991 (4.5%)	396 (2.7%)	974 (4.5%)
3	101 (0.6%)	248 (1.1%)	84 (0.6%)	244 (1.1%)
>=4	48 (0.3%)	101 (0.5%)	29 (0.2%)	97 (0.4%)

Baseline Characteristics -- for older population (>=60) available at baseline and after 90 days

	Population at baseline		Population after 90 days	
	C19 (N=3447)	vaccination (N=14807)	C19 (N=1998)	vaccination (N=13773)
**age**				
Mean (SD)	71.6 (9.51)	77.5 (11.6)	66.9 (6.37)	77.0 (11.6)
Median [Q1,Q3]	69.0 [63.0,78.0]	78.0 [66.0,87.0]	65.0 [62.0,70.0]	77.0 [65.0,87.0]
**factor(sex)**				
Female	1758 (51.0%)	10094 (68.2%)	977 (48.9%)	9442 (68.6%)
Male	1689 (49.0%)	4713 (31.8%)	1021 (51.1%)	4331 (31.4%)
**factor(comorbidity_count)**				
0	1072 (31.1%)	2606 (17.6%)	826 (41.3%)	2584 (18.8%)
1	916 (26.6%)	2184 (14.7%)	606 (30.3%)	2122 (15.4%)
2	617 (17.9%)	3027 (20.4%)	305 (15.3%)	2795 (20.3%)
3	429 (12.4%)	3313 (22.4%)	167 (8.4%)	3017 (21.9%)
>=4	413 (12.0%)	3677 (24.8%)	94 (4.7%)	3255 (23.6%)

Table ST2 Results of different regression analysis infection is considered as reference (compare with illustration in Figure 4 in manuscript). (HR hazard ratio, OR odds ratio)

Outcome:		C19		death	
population		HR	OR	HR	OR
overall	**unadjusted**	3.127 [2.016;4.851]	3.116 [2.052;4.961]	16.12 [10.087;25.762]	16.218 [10.473;26.916]
	**adjusted**	3.464 [2.194;5.469]	3.471 [2.24;5.614]	1.609 [0.982;2.637]	1.613 [1.008;2.745]
	**Matched**	1.867 [0.871;4.002]	1.87 [1.308;2.674]	0.827 [0.512;1.337]	0.833 [0.699;0.993]
	**Weighted**	2.295 [1.255;4.2]	2.294 [2.921;0.004]	1.956 [1.025;3.732]	1.96 [2.244;0.012]
<60	**unadjusted**	4.247 [2.544;7.09]	4.254 [2.621;7.357]	2.064 [0.417;10.224]	2.059 [0.474;14.053]
	**Adjusted**	4.217 [2.499;7.116]	4.225 [2.571;7.378]	1.955 [0.394;9.691]	1.977 [0.435;13.85]
	**Matched**	4.701 [2.77;7.98]	4.847 [2.642;8.893]	2.519 [0.489;12.987]	5.195 [3.482;7.751]
	**Weighted**	4.371 [2.548;7.497]	4.378 [6.178;0.005]	1.474 [0.283;7.667]	1.47 [4.227;0]
>=60	**unadjusted**	1.099 [0.469;2.576]	1.088 [0.502;2.849]	5.509 [3.353;9.052]	5.613 [3.532;9.641]
	**Adjusted**	0.966 [0.389;2.399]	0.966 [0.414;2.646]	1.575 [0.941;2.637]	1.589 [0.975;2.781]
	**Matched**	0.577 [0.253;1.316]	0.544 [0.318;0.93]	0.772 [0.481;1.238]	0.544 [0.318;0.93]
	**weighted**	0.801 [0.299;2.143]	0.797 [1.171;0.003]	1.761 [0.916;3.385]	1.781 [2.044;0.04]
